# Exposure of children to multiple long-term air pollutants (PM_2.5_, BC, NO_2_) and noise at home and school in accra, Ghana

**DOI:** 10.1088/1748-9326/ae8d40

**Published:** 2026-09-01

**Authors:** Rachel Hulme, Iris EM Fynn, Abosede S Alli, Kate A Kyeremateng, Carissa L Lange, Barbara E Mottey, Majid Ezzati, Allison F Hughes, Raphael E Arku, Sierra N Clark

**Affiliations:** 1 School of Health and Medical Sciences, City St. George’s, University of London, London, United Kingdom; 2 Department of Geography and Resource Development, University of Ghana, Legon, Accra, Ghana; 3 Department of Environmental Health Sciences, University of Massachusetts, Amherst, MA, United States of America; 4 Department of Biostatistics and Epidemiology, University of Massachusetts, Amherst, MA, United States of America; 5 Department of Epidemiology and Biostatistics, School of Public Health, Imperial College London, London, United Kingdom; 6 Regional Institute for Population Studies, University of Ghana, Accra, Ghana; 7 MRC Centre for Environment and Health, School of Public Health, Imperial College London, London, United Kingdom; 8 Imperial Global Ghana, Accra, Ghana; 9 Department of Physics, University of Ghana, Legon, Accra, Ghana

**Keywords:** air pollution, noise, children, Sub-Saharan Africa, school

## Abstract

Sub-Saharan African (SSA) cities have high air and noise pollution levels, yet limited information exists on children’s joint exposures at home and school locations - the two most important environments where children spend much of their time. This study characterised schoolchildren’s exposure to multiple air pollutants and environmental noise and sound sources within the Accra School Health and Environment Study (ASHES). ASHES involved 1034 children aged 8–12 years from 90 public (73%) and private schools in Accra, Ghana, a major SSA city. Annual mean concentrations of fine particulate matter (PM_2.5_), nitrogen dioxide (NO_2_), and black carbon (BC), as well as environmental noise (*L*_day_, *L*_night_, *L*_den_) and sound sources, were derived from land use regression models and linked to geocoded school and home locations for 919 children with valid data. A time-location weighted total exposure estimate was calculated for the air and noise pollution metrics. We also examined children’s responses to a noise annoyance survey in relation to their noise exposure levels at home. For air pollutants, median home exposures were 33.8 *μ*g m^−3^ (PM_2.5_), 55.5 *μ*g m^−3^ (NO_2_), and 5.2 × 10^−5^m^−1^ (BC), and corresponding median school exposures were 29.9 *μ*g m^−3^, 53.5 *μ*g m^−3^, and 4.9 × 10^−5^m^−1^. The time location-weighted total air pollutant exposures for most children surpassed the respective World Health Organization (WHO) guideline. Similarly, median environmental noise levels (in dBA) at home (*L*_den_ = 66.3 and *L*_night_ = 54.2) and at school (*L*_day_ = 62.4) exceeded WHO thresholds for road-traffic noise and Ghana’s noise standard for areas with educational facilities, respectively. Pollution levels at home and school were moderately correlated (r [PM_2.5_] = 0.56; r [NO_2_] = 0.33; r [*L*_den_]= 0.47). On average, children attending public schools or living in lower income areas had higher exposures to both air and noise pollution than their private school (fee-paying) or higher-income counterparts. In contrast, nature-based sounds were more common in higher income areas. Among children exposed to *L*_den_ noise levels between 65 and 70 dBA, 35% reported being highly annoyed to road traffic noise. Schoolchildren in Accra experience air and noise pollution levels exceeding health-based guidelines and standards. Exposures are unequally distributed, highlighting environmental inequalities with implications for child health, development, and learning.

## 1. Introduction

Places where children live, play, and learn critically shape their health and development. In sub-Saharan Africa (SSA), urban children enjoy better access to healthcare, education, nutrition, technology, and other essential serviches than their rural peers [1]. However, urban children also face challenges such as exposure to poor environmental conditions, including outdoor air and noise pollution. Unlike cities in high-income countries, where patterns of air and noise pollution exposures are primarily related to industrial and transportation emissions, sources of urban air and noise pollution in SSA are diverse [2]. These include increasing traffic congestion [3], high prevalence of biomass use for both household and commercial activities [4], indiscriminate trash and electronic waste burning, small-scale/informal industrial activities and diesel generator use in residential areas, and loud public music from various sources (e.g. small-scale business and shops, and religious activities) [5]. All these significantly influence pollution levels and patterns, which in turn may affect children’s exposure and health.

Children’s exposure to air pollution has been linked with adverse lung and respiratory outcomes [6] (e.g. asthma exacerbations) [7] as well as sub-clinical cardiometabolic indicators [8] (e.g. raised blood pressure, and overweight), and exposure to both air and environmental noise pollution have been shown to affect sleep [9], and behaviour and cognitive development [10]. These exposures can further influence school attendance (absences) and performance (e.g. comprehension, numeracy, test scores and overall grade point averages) [11], with implications across the life course for educational attainment and socioeconomic empowerment in adulthood [12]. Yet, much of this research has been conducted in high-income countries, where emission sources are less diverse and exposure levels are, on average, lower and narrower in range. There remains a significant gap in our understanding of children’s exposures in important microenvironments in fast-growing SSA cities. Even less is known about their effects on child health.

School-aged children spend a significant portion of their time at home and school, making these two microenvironments primary places of exposure to air and noise pollution. In SSA context, spatial inequalities in household and neighbourhood socioeconomic factors within cities can further exacerbate small-area disparities in environmental exposures thereby potentially widening health inequalities. Because many SSA cities still lack the infrastructure and policy frameworks necessary to monitor, enforce and thus mitigate pollution exposures [13], there is a pressing need for data and evidence on children’s exposure to environmental pollutants, particularly in locations where they spend most of their time [14]. Such data can inform targeted public health interventions and policies that protect children’s health and reduce health inequalities in SSA cities.

In Accra, Ghana’s capital and largest city, air pollution and environmental noise have become the focus of increasing monitoring, modelling, and research efforts. Two decades of combined data from the Greater Accra Metropolitan Area (GAMA) indicate consistently high and spatially heterogeneous levels of both exposures, driven by a complex mix of shared sources, such as road traffic, and source-specific contributors, including household biomass use and amplified music from religious institutions and small businesses [5, 15, 16]. Despite this growing evidence base, no study has simultaneously characterised children’s exposure to both air pollution and environmental noise at home and school in a sub-Saharan African city, the two microenvironments where they spend most of their time. Limited evidence suggests that schoolchildren in Accra experience high personal air pollution exposures and that factors such as proximity of schools to major roads, nearby biomass-burning activities, and school ground surface characteristics contribute substantially to these exposures [17]. In a prior study in the GAMA, we collected short-term (week-long) air and noise pollution measurements within 90 elementary schoolyards and found that none of the schools met health-based air quality guidelines, and fewer than one-third complied with recommended national noise limits for educational settings [18]. However, this work did not assess exposures at children’s homes, another critical microenvironment likely shaped by similar spatial and socio-economic factors, nor did it capture long-term air pollution or noise exposure at the school locations. Characterizing children’s environmental exposures in SSA’s complex urban settings therefore requires long-term data across both school and residential microenvironments.

In this paper, we linked location information from participants in the Accra School Health and Environment Study (ASHES) with high-resolution geocoded air and noise pollution and sound source data and characterised inequalities in children’s school, home, and total pollution exposures in the GAMA. ASHES is a cross-sectional study designed to evaluate air and noise pollution at elementary schools and for schoolchildren, and their influence on key markers of childhood health and development in an urban SSA context [19].

## 2. Methods

### 2.1. Study setting

The GAMA is a densely populated urban region with approximately 6 million residents in its 250 km^2^ area and an annual population growth rate of 4.2% [19]. GAMA comprises 25 districts, including the more urbanized Accra metropolitan area (AMA) and the port city of Tema (TMA). The metropolis exhibits significant socioeconomic disparities income, housing, and environmental conditions, and varies substantially by population density, land use, and urbanity [20, 21]. Public transportation in the GAMA is dominated by privately owned minibuses (‘trotro’), taxis, and emerging ride-share and motorcycle taxi services, with limited train system and formal transit options [18, 22]. Weak urban planning and regulations coupled with rapid expansion have contributed to a changing landscape of sources of air pollution [19]. Accra’s tropical climate, with average temperatures and relative humidity between 25 °C–33 °C (77–90°F) and 77%–85%, respectively, further influences environmental exposures, particularly during the Harmattan season when dust-laden winds exacerbate air quality issues [23]. Collectively, these characteristics of the urban environment are likely to influence children’s cumulative exposure to air pollution and environmental noise, with potential consequences for their health and development.

In Ghana, social and environmental inequalities are strongly associated with child health outcomes, including child mortality [24, 25]. Recent analyses have also shown nearly five-fold variation in under-five mortality across neighbourhoods within GAMA, with the highest rates observed in the densely populated urban core and industrial areas, which also experience some of the highest levels of environmental pollution [22, 25]. Preliminary findings from our ongoing ASHES study indicate a substantial burden of adverse health outcomes among schoolchildren: approximately one-third have elevated blood pressure, 30% are overweight or obese, and 15% exhibit behavioural problems [19]. Emerging evidence from these analyses (with several manuscripts currently at various stages of submission) suggests that environmental pollution may be an important contributor to these health burdens.

### 2.2. Data sources

#### 2.2.1. The ASHES

Details of the ASHES aims, design, school selection, and data have been described previously [19]. Between July 2022 to May 2023, ASHES recruited 1037 children aged 8–12 years from 90 (27% private) elementary schools selected from each of GAMA’s metropolises and municipalities. Public schools were over-represented as that is where national and local policy interventions are implemented and enforced. Of the 1037 children recruited, three lived outside the study area and homes and schools of the remaining 1034 were geocoded. For all participants, we gathered information on a wide range of individual demographic, health and household characteristics using structured interviewer-administered surveys.

#### 2.2.2. Modelled air pollution, noise and sound sources

We leveraged statistical land use regression (LUR) models recently developed for the GAMA to estimate long-term exposures to air pollution and environmental noise. The air pollution models provided estimates of annual average concentrations of fine particulate matter (PM_2.5_, *μ*g m^−3^), black carbon (BC, 10^−5^m^−1^), and nitrogen dioxide (NO_2_, *μ*g m^−3^). BC, a component of PM_2.5_, was measured as the optical absorption coefficient, which quantifies the amount of light absorbed by particles collected on the PM_2.5_ filters [26]. It is typically obtained via reflectance measurement with a smoke-stain reflectometer and expressed in units of 10^−5^m^−1^ (i.e. one absorbance unit = 1 × 10^−5^m^−1^ absorption coefficient). This metric was developed for and validated in the ESCAPE study across 20 European areas as a filter-based proxy for BC/soot, particularly suited to spatial exposure modelling [27]. Following the calibration reported by Jeronimo *et al* [26], one absorbance unit corresponds to approximately 1.67 *μ*g m^−3^ elemental carbon (EC). For interpretability, BC was scaled per 10 × 10^−5^ m^−1^, representing a realistic range of exposure.

The environmental noise and sound source models generated estimates of annual equivalent continuous sound levels, including *L*_den_, *L*_night_, and *L*_day_ (measured in A-weighted decibels [dBA]), as well as novel metrics describing the prevalence (%) of specific sound types and sound sources (e.g. road traffic and bird sounds) throughout the day and night.

The spatial LUR models were developed using data from a comprehensive measurement campaign conducted across GAMA during 2019–2020, which included ten fixed monitoring sites operating continuously for one year and 136 rotating sites sampled over week-long periods. Monitoring locations were selected using a stratified random design that accounted for population density and land-use characteristics. Predictor variables included road traffic, land use, population and building density, vegetation, elevation, points of interest (including the international airport), and time-varying meteorological conditions. Model performance was evaluated using repeated 10-fold cross-validation. Cross-validated *R*^2^ values ranged from 0.58 to 0 · 83 for PM_2.5_ and from 0.79 to 0.88 for BC across Harmattan and non-Harmattan periods. The annual NO_2_ model achieved an *R*^2^ of 0.80, whereas environmental noise models yielded *R*^2^ values of 0.51–0.54 across daytime and nighttime periods. Models for sound-source prevalence (%) demonstrated moderate-to-good performance, with correlation coefficients (r) ranging from 0.68–0.69 for road-traffic sounds and from 0.59–0.72 for animal and insect sounds.

The air pollution estimates were generated at 50 m spatial and weekly temporal resolutions over the entire GAMA, whereas environmental noise estimates were generated at the same spatial resolution for different periods of the 24 h day. Detailed descriptions of the measurement campaign, exposure models, and validation procedures have been published previously [5, 21]. Further details of the LUR models, including model development and validation can be found here [21, 22, 28]. Additional information on environmental noise metrics and sound-source classification is provided in the Supplemental Information (SI1).

#### 2.2.3. Area-level socioeconomic status (SES)

Area-level SES was assessed using median log-equivalised household consumption (Ghanaian Cedi [GH₵]) at the census enumeration area (EA) level, the smallest census administrative unit in Ghana (median population approximately 800). Household consumption estimates were derived using small-area estimation methods [29, 30]; that combined data from the 2012–13 Ghana Living Standards Survey (GLSS6; 93% response rate) with the 100% sample of the 2010 national census. Total annual household consumption was calculated from expenditures on food, housing, utilities, transportation, education, health care, and other goods and services, as well as rental value. Small-area estimation models were then used to predict household consumption for all EAs based on census-derived information on asset ownership, education, employment, housing characteristics, and sociodemographic factors. The median log-equalised household consumption for each EA was used as the measure of area-level SES. Additional details on the SES modelling approach have been reported previously [28]. Amongst the ASHES cohort, residential neighbourhood SES (median log-equivalised household consumption) was fairly normally distributed, with a median value of 8.828 (IQR: 8.670–8.946), and a range from 7.987 to 9.746.

#### 2.2.4. Data linkage

The schools and homes of the ASHES participants were geocoded and linked with the spatial air and noise pollution and sound sources prediction model maps, along with socioeconomic data, to estimate concentrations and SES at the different microenvironments for each child. Due to missing location information for some children (115 had incomplete survey responses and 3 lived outside of GAMA), we linked data for 919 of the 1034 eligible children (89%). The 118 children with missing data were distributed across 28 of the 90 schools (average of 2 missing children per school), suggesting missingness was not concentrated in a small number of schools or locations. Mean age among children with missing data (10.7 years) was comparable to the analytic sample, and missingness was primarily attributable to incomplete survey administration rather than any known systematic factor related to residential location or exposure. These patterns support the assumption that data were missing at random.

### 2.3. Derived total exposure metrics

We constructed a total exposure metric for each child using time-weighted average measures of air and noise pollution derived from the home and school and information on time children spend at both locations per day and across days of the week collected as part of the ASHES survey (table 1). We did not have information on time that children spent in transit and away from home or school or modes of transport. However, based on the home and school geocoded information, ∼80% of the children lived within 1 km of the school, thus, we assumed that estimates in these two key microenvironments generally reflect their overall exposures. We also did not derive a total exposure metric for night-time noise levels as it was assumed that children spend their nights at home only.

**Table 1.** Weights used to derive a time-location average measure of total exposures.

Exposure	School weight	Home weight
Annual average air pollution concentration data (PM_2.5_, BC, NO_2_)	6–8 h^a^ × 5 d in a week (weekdays) + 0 h × 2 d in a weekend Weight range: 30–40	16–18 h^a^ × 5 d in a week (weekdays)+ 24 h × 2 d in a weekend Weight range: 128–138
Annual average day-time environmental noise exposure (*L*_day_)^b^	5 d in a week (weekdays) Weight: 5	2 d in a weekend Weight: 2

^a^ Specific to each school’s reported hours in the ASHES survey.

b Since *L*_day_ already reflects a specific time of day, the weighting in this total exposure metric is entirely based on the number of days children spend at either home or school during the daytime.

### 2.4. Descriptive analyses

We estimated medians, interquartile ranges, and percentages (*n* [%]) to descriptively summarise the overall levels of air and environmental noise pollution at schools and homes of the schoolchildren, along with their total exposures. The estimated levels were compared against international guidelines and national standards, where relevant. The correlation between household- and school-level exposures of the same pollutant type, as well as across pollutant types within same microenvironment, were investigated using linear (Pearson, r) correlations and visually for non-linearity (scatter plots and splines). We also explored potential inequalities in exposures between children attending public or private schools as well as school- and home-specific area-level SES, with descriptive statistics and non-parametric tests (Wilcoxon Rank Sum Test) of difference in the distributions. Finally, we used students’ survey responses to calculate a quantitative metric describing the percentage of students ‘highly annoyed’ (HA) by road-traffic noise at their home location. Students reporting numerical values of 8 or above from 11-point numeric questions, were coded as being ‘HA’ [1] while values of 7 or lower responses were coded as being ‘not highly annoyed’ (0). This is consistent with ISO guidelines (ISO/TS 15666:2021) and general convention within the noise and health literature. Previous ASHES analysis of annoyance at schools confirmed the agreement in responses when asked as a numerical versus a verbal rating scale [18]. We created and plotted exposure-response curves to represent the unadjusted relationship between increasing levels of modelled noise exposure at children’s homes (*L*_den_, *L*_night_) and the proportion of children within each of those noise level bands reporting being HA to road-traffic noise [31]. These exposure-response curves are unadjusted for other covariates.

### 2.5. Ethical approval

The ASHES study was approved by the Institutional Review Boards at the lead institutions: the University of Massachusetts Amherst and the University of Ghana, Legon. Additional research authorizations within schools were secured across various administrative levels of the Ghana Education Service (GES), including from heads of participating schools. A research member at the University of Ghana conducted the geographic linkages of pollution maps and home and school locations, and an anonymised dataset was produced for this analysis.

## 3. Results

### 3.1. Spatial distribution and levels of pollution exposures

For all children in the cohort, predicted annual PM_2.5_ and NO_2_ levels at both home and school locations exceeded the World Health Organization (WHO) annual guideline of 5 ug m^−3^ and 10 ug m^−3^, respectively. At residential locations, annual PM_2.5_ concentrations ranged from 27.2–40.7 ug m^−3^ and NO_2_ concentrations from 13.1–108.8 ug m^−3^. At school locations, PM_2.5_ concentrations ranged from 26.3–35.4 ug m^−3^ and NO_2_ concentrations from 23.1–116.8 ug m^−3^ (table 2). Similarly, environmental noise levels (*L*_den_ and *L*_night_) at home locations of each child were above WHO guidelines of 53 dBA *L*_den_ and 45 dBA *L*_night_ for road-traffic noise exposure (table 2) [32]. Ninety-eight percent (98%) of the schools had day-time (6:00–22:00) environmental noise levels (*L*_day_) above 55 dBA, Ghana’s standard for areas with educational and hospital facilities. On average, the levels of air pollution and environmental noise were slightly higher at the children’s homes than at schools.

**Table 2.** Model estimated air pollution concentrations, environmental noise levels, and other indicators of the urban sound environment, at Accra children’s home and school locations, and total time-weighted estimates.

	Total Exposure			Home			School		
Summary measure	Median	IQR	Range	Median	IQR	Range	Median	IQR	Range
**Air pollutants**									
*PM_2.5_ (μ*g m^−3^)	33.7	32.1–35.2	27.7–39.9	33.8	31.9–35.4	27.2–40.7	29.9	28.7–31.6	26.3–35.4
*BC* (10^−5^m^−1^)	5.4	4.7–6.0	3.3–12.9	5.2	4.6–6.0	3.1–15.11	4.9	4.3–5.7	3.3–8.9
*NO_2_* (*μ*g m^−3^)	54.4	45.3–62.6	16.2–98.2	55.5	45.1–64.1	13.1–108.8	53.5	43.5–62.0	23.1–116.8

**Environmental noise** (dBA)									
*L*_den_	—	—	—	63.9	61.0–67.0	54.0–87.4	—	—	—
*L*_night_	—	—	—	54.2	51.6–57.2	45.8–80.0	—	—	—
*L*_day_	62.2	59.5–64.9	54.2–71.2	62.6	59.6–65.8	51.2–84.0	62.4	59.4–64.7	54.7–70.6

**Sound sources** (% of time present)									
*Day-time Road-traffic sounds*	—	—	—	74.5	65.1–79.3	41.2–88.3	74.9	66.5–79.2	50.0–86.8
*Night-time Road-traffic sounds*	—	—	—	53.4	40.1–61.3	21.2–79.6	—	—	—
*Day-time Animal sounds*	—	—	—	32.5	20.9–47.8	9.8–64.6	32.2	22.9–44.8	12.9–61.9
*Night-time Animal sounds*	—	—	—	50.2	39.9–62.2	20.7–80.5	—	—	—

Legend: *L*_den_: Day-Evening-Night Equivalent continuous sound level; *L*_day_: Day-time sound level; *L*_night_: Night-time sound level; PM_2.5_: Fine particulate matter; NO_2_: Nitrogen dioxide; BC: Black carbon.

**Figure 1.**
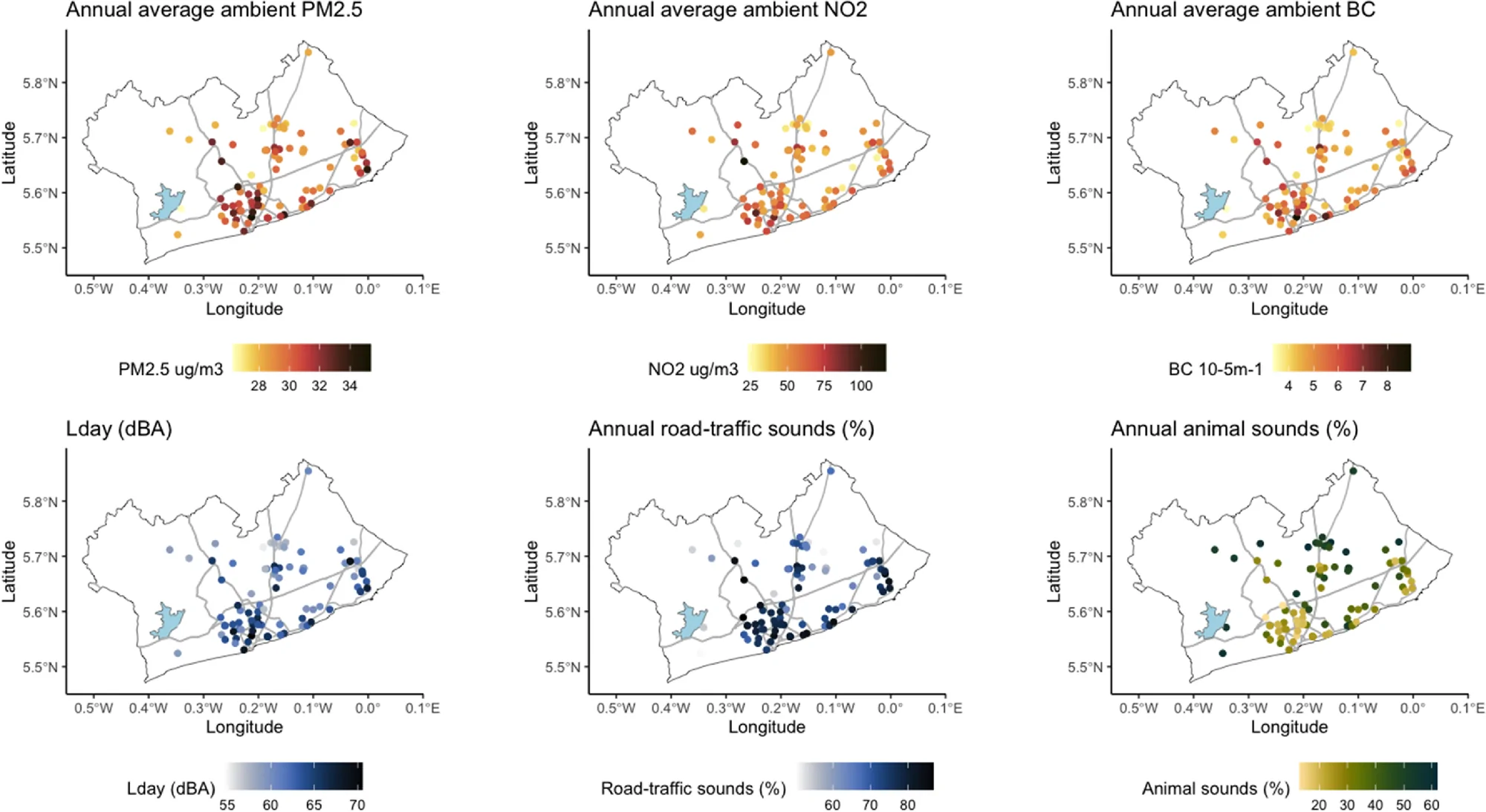
Annual average air pollution concentrations, day-time environmental noise levels, and sound type metrics at ASHES project schools across the Greater Accra Metropolitan Area (GAMA). Note the varying colour scales between the maps. Units for BC are 10^−5^ m^−1^, where 1 absorbance unit is equivalent to 1.67 ug m^−3^ EC.

There was considerable spatial variability in several pollutants between home and school environments (table 2). Schools located in the more densely populated and urbanised area of the AMA experienced higher median NO_2_ concentration (58 ug m^−3^) and noise levels (median *L*_den_: 64 dBA) compared to their more geographically dispersed northern and peri-urban counterparts (figure 1). Similar trends were observed for other pollutants at home locations as well. Median NO_2_ levels in AMA were 62 ug m^−3^ compared with 55.5 ug m^−3^ and median noise levels (*L*_den_) were at 66 dBA compared with 64 dBA across wider GAMA (table 1). However, the reverse spatial trend was true for the distribution of nature-based animal and insect sounds near schools and homes.

### 3.2. Relationship between children’s exposures at home and at school

There is substantial spatial co-occurrence of air pollution and traffic-related noise exposures across both home and school environments. At residential locations, air pollution and noise exposures were highly correlated, with pairwise Pearson correlation coefficients among PM_2.5_, NO_2_, BC, *L*_den_, *L*_night_, and nighttime road-traffic noise prevalence ranged from 0.79–0.99. Similarly, at school locations, moderate-to-strong correlations were observed among PM_2.5_, NO_2_, BC, *L*_day_, and daytime road-traffic noise prevalence, with correlation coefficients ranging from 0.60–0.90.

**Figure 2.**
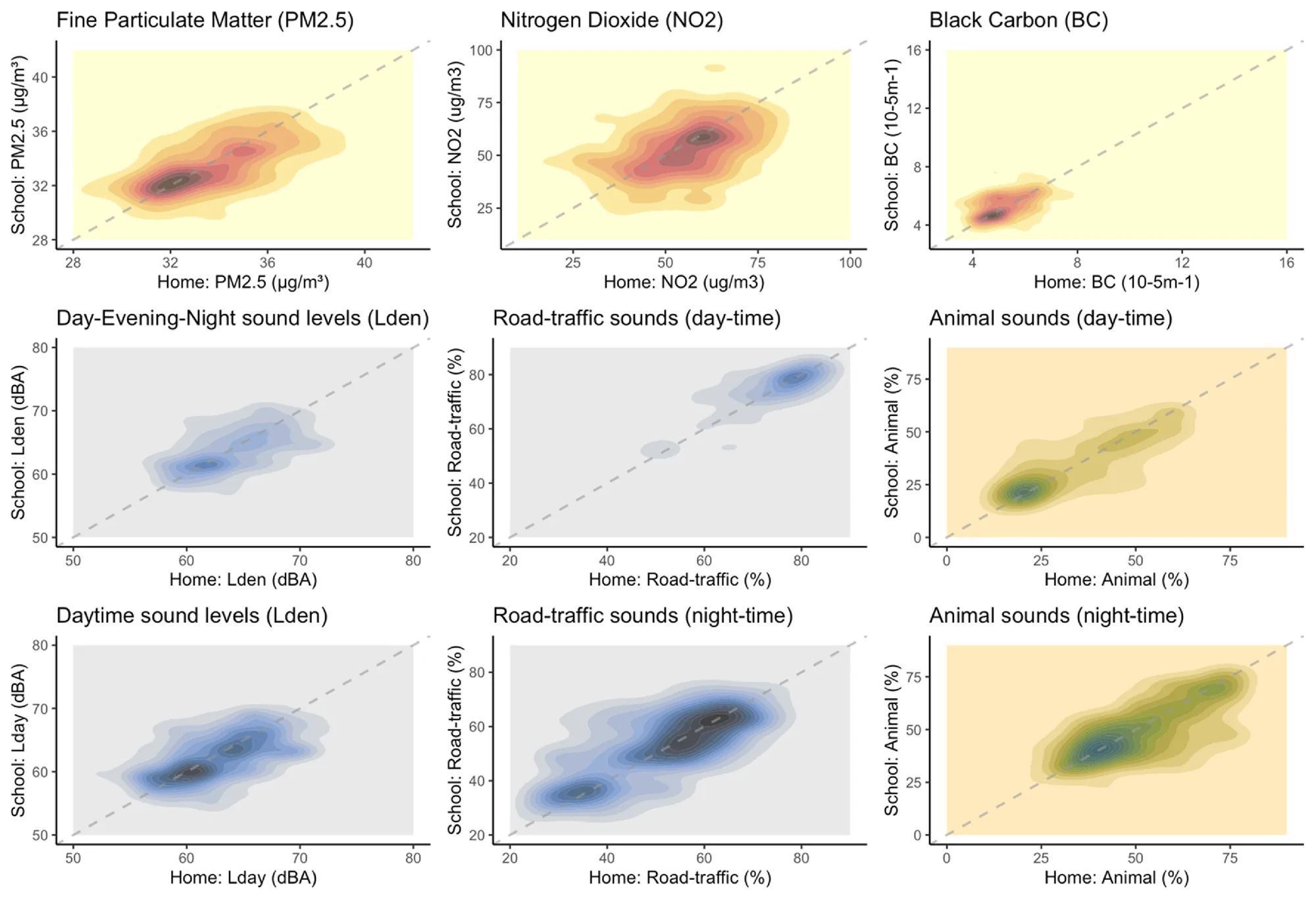
Relationship between home and school air pollution concentrations, environmental noise levels and sound type metrics for children in the ASHES cohort. The shading indicates the density of the correlation between school and home exposure levels for children, with darker shades corresponding to a higher density of data points and vice versa. The 45-degree line indicates what would be perfect correlation in the data (Home vs School exposures). Pearson correlation coefficients (r) for PM_2.5_ = 0.56, NO_2_ = 0.33, BC = 0.34, *L*_den_ = 0.47, *L*_day_ = 0.50, Road-traffic sounds (day) = 0.65, Road-traffic sounds (night) = 0.68, Animal sounds (day) = 0.71, Animal sounds (night) = 0.67.

When comparing the same pollutant across home and school locations, correlations were generally moderate to weak, with Pearson correlation coefficients of 0.56 for PM_2.5_, 0.33 for NO_2_, and 0.34 for BC (figure 2). For BC in particular, some children experienced substantially higher exposures at home (>12 × 10^−5^ m^−1^) than at school, suggesting that important emission sources may differ between residential and school environments. In contrast, environmental noise metrics and sound-source prevalence showed stronger correspondence between home and school locations, with correlation coefficients ranging from 0.47–0.71 (figure 2). For example, the median prevalence of daytime road-traffic sounds was nearly identical at home and school locations (74.5% vs 74.9% of time present), as was the prevalence of animal and insect sounds (32.5% vs 32.2%). Thus, although the children’s air pollution exposures differ substantially between home and school environments, their soundscape characteristics are generally more similar across these settings.

### 3.3. Children’s total exposures to air pollution and environmental noise

The median annual average total (combined home and school) exposure to PM_2.5_ was 33.7 *µ*g m^−3^ (IQR: 32.1–35.2 *µ*g m^−3^), with exposures for all children exceeding the WHO annual guideline of 5 *μ*g m^−3^. Similarly, median annual average NO_2_ total exposure was 54.4 *µ*g m^−3^ (IQR: 45.3–62.6 *µ*g m^−3^), with all estimates surpassing the WHO guideline of 10 *µ*g m^−3^. The median total exposure for environmental noise during the day-time (*L*_day_) was 62.2 dBA (IQR: 59.5–64.9 dBA).

### 3.4. Socioeconomic inequalities in exposures

**Figure 3.**
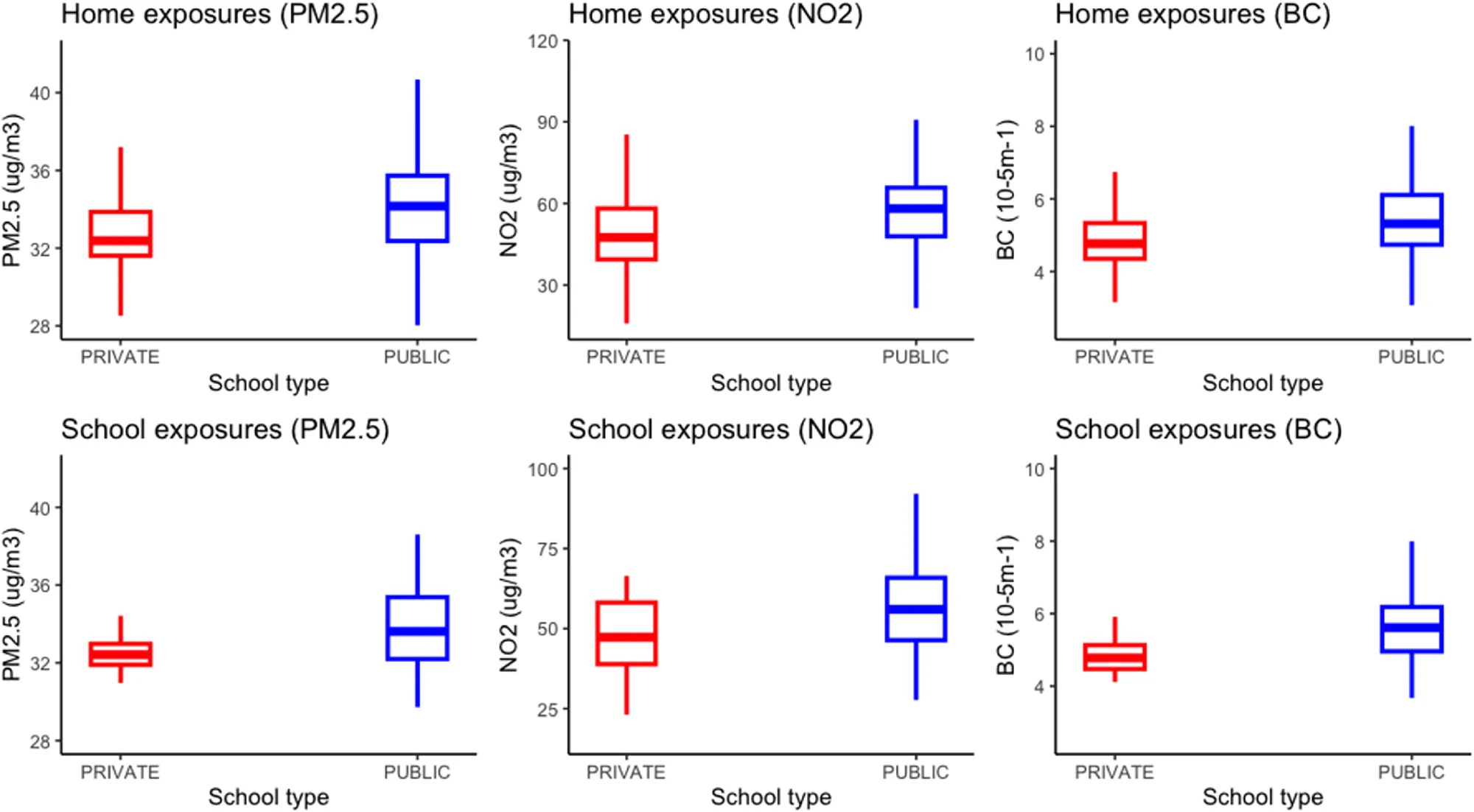
Box and Whisker plots showing air pollution levels at homes and schools, distributed across students who go to public and private schools. Box and whisker plots represent the mean and interquartile range for each dataset. Units for BC are 10^−5^m^−1^. The distribution in private schools differs significantly (*p* < 0.01) than the distribution in public schools for all air pollution concentrations using non-parametric tests of difference.

**Figure 4.**
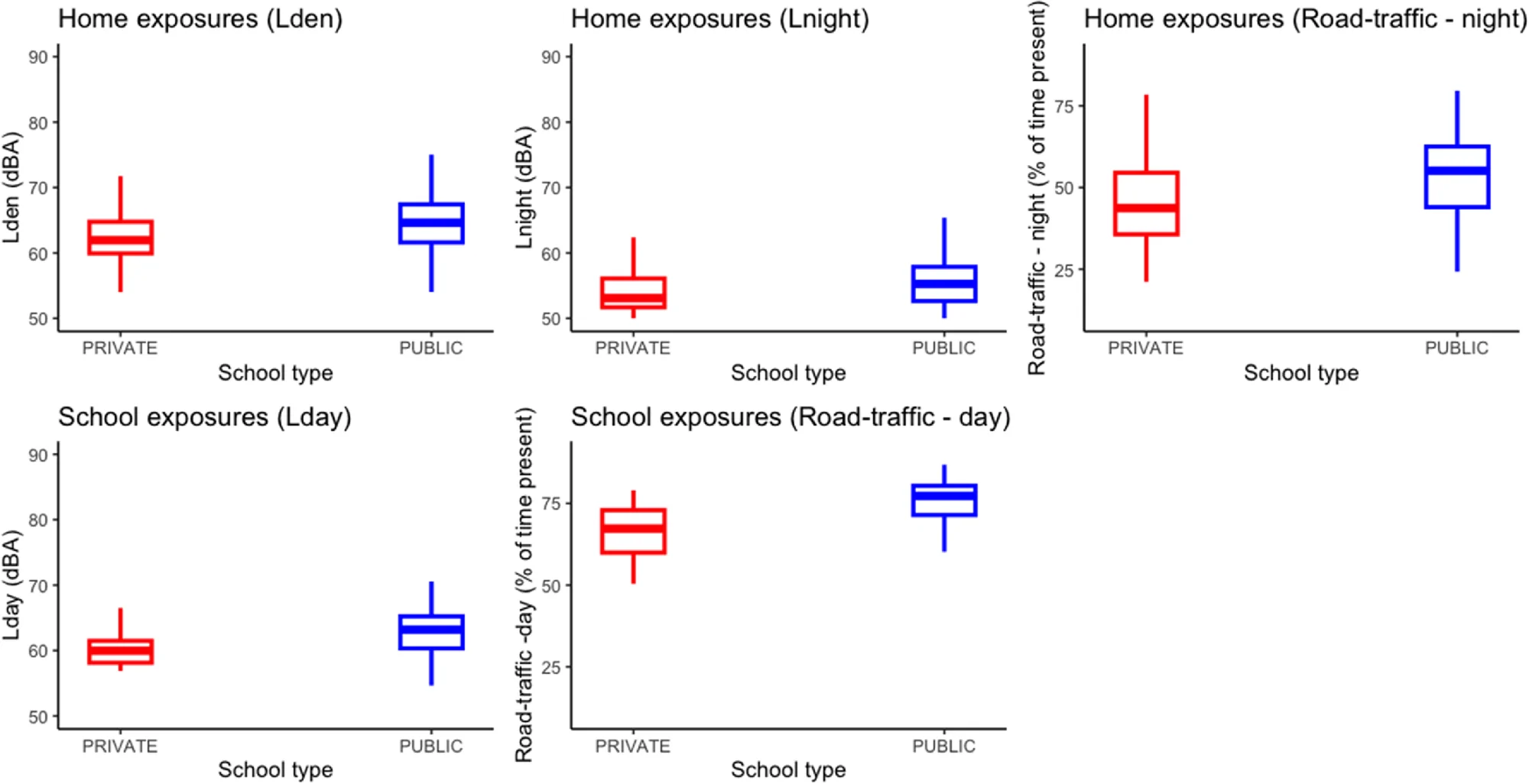
Box and Whisker plots showing environmental noise levels (dBA) and road-traffic sounds (%) at homes and schools, distributed across students who go to public and private schools. Box and whisker plots represent the mean and interquartile range for each dataset. The distribution in private schools differs significantly (*p* < 0.01) than the distribution in public schools for all noise exposures using non-parametric tests of difference.

The children’s exposures differed by school types (private vs public). On average, children attending private schools had lower levels of both air and environmental noise pollution compared to those in public schools (figures 3 and 4), though the magnitude of the differences varied by pollutant. The largest differences (∼22%) in median levels were observed for NO_2_ (56.0 vs 45.8) and BC (5.2 vs 4.2) concentrations, whereas PM_2.5_ (30.6 vs 28.8) and *L*_day_ (63 vs 60) recorded the smallest differences. Using Wilcoxon rank-sum test, the distribution of all air pollution and environmental noise exposure metrics differed significantly between public and private schools (all *p* < 0.01). Nonetheless, there were substantial variations with significant overlap in pollutant level distributions around the median value within each school type. The same patterns persisted at the children’s home locations, where NO_2_ levels at homes of those attending public schools were ∼22% higher than for those in private schools.

**Figure 5.**
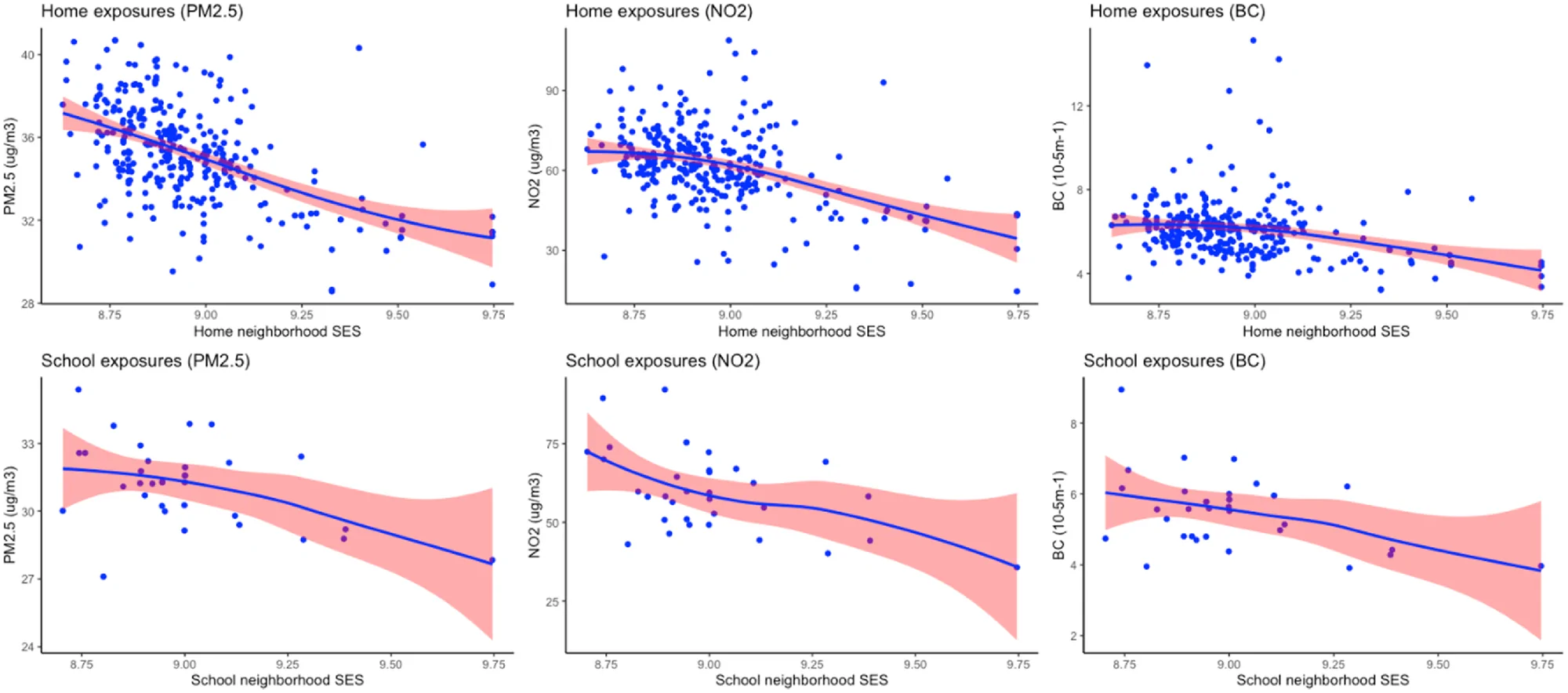
Scatter plots and loess smoothed line graphs of the correlation between home and school neighbourhood socioeconomic status (SES) and air pollution levels within the Accra Metropolis (AMA). Home and school location air pollution levels include PM_2.5_, NO_2_ and BC.

Additionally, children living in the urbanized city core of AMA and going to school in neighbourhoods with lower SES on average experienced higher air and environmental noise pollution at school locations than their counterparts (figure 5). For example, children living in the lowest SES neighbourhoods (bottom 33%) had higher median PM_2.5_ (36.7 vs 34.5 *µ*g m^−3^), NO_2_ (69 vs 58.3 *µ*g m^−3^), BC (6.6 vs 5.7 × 10^−5^m^-^), and (*L*_den_: 69.7 vs 65.1 dBA) than children living in the highest (upper 33%) SES areas. This trend was also observed for the home environment, with children living in lower SES EAs having higher levels of exposure (figure 5).

### 3.5. Annoyance to road-traffic noise

In unadjusted analyses, the share of the children who reported being highly annoyed to road-traffic noise at home increased with increasing levels of environmental noise (*L*_den_) predicted at their homes (figure S1 in SI 2). Amongst children exposed to *L*_den_ <60 dBA, only 20% reported being HA to road-traffic noise, compared >35% among those exposed to *L*_den_ >65 dBA. A similar pattern was observed for night-time noise.

## 4. Discussion

Despite growing evidence of high and spatially heterogeneous levels of air and environmental noise pollution in rapidly urbanising cities across SSA, no study has simultaneously characterised children’s exposure to both pollutants in the home and school environments, the two microenvironments where they spend most of their time. We leveraged consistent and comparable high-resolution geocoded air and noise pollution and sound source data to examine the levels and inequalities in schoolchildren’s school, home, and total exposures in Accra, Ghana, a major SSA city where increasing urban and economic growth are influencing environmental pollution but there is limited data on the exposures that children face. We found that nearly all schoolchildren in the study were living and learning in environments where noise and air pollution levels far exceeded both local standards and international health-based guidelines. The children’s air pollution and traffic-related noise exposures showed substantial spatial co-occurrence across both home and school environments. However, home–school correlations were stronger for environmental noise and soundscape characteristics than for air pollutants, indicating greater variability in children’s air pollution exposures across settings than in their acoustic environments. On average, children attending public schools or living in lower SES neighbourhoods experienced higher levels of air and environmental noise pollution and road-traffic sound at home and at school. The proportion of children annoyed by road-traffic at home rose with increasing noise levels.

There were moderate correlations between our prior 7 day schoolyard measurement study [18] and the current model-based estimates at the school locations (Pearson r ranged 0.37–0.44 for *L*_day_ noise and BC air pollution). Several factors likely contributed to the moderate correlation between measured and modelled values. First, the modelled exposures represent spatially averaged estimates based on predictor variables, whereas measurements were collected at fixed points and likely more sensitive to local/nearby features (e.g. nearby sources). Second, intermittent and event-driven sources could have introduced variability in the schoolyard measurement that was poorly captured by average-based estimates. Third, it was also possible that the final city-wide LUR models might be missing some predictor (s), such as traffic flow vehicle/volume and fleet composition. With the modelled data, we were able to compare both home and school environments and in addition developed aggregated exposure metrics for each child. Yet still, the current findings, particularly the spatial and socioeconomic disparities in exposure, are consistent with the prior study, and both sources of data provided consistent information about schoolchildren’s exposure in a major fast-evolving SSA city.

Our findings also align with global patterns of high urban air pollution and environmental noise exposures, particularly in low- and middle-income countries (LMICs), where the levels frequently exceed national and international policy and regulatory targets, both for adults and children [33, 34]. Cities in SSA have some of the highest recorded concentrations of PM_2.5_ globally [35]. Average PM_2.5_ levels in SSA cities are estimated at around 100 *µ*g m^−3^, compared to <20 *µ*g m^−3^ typically found in most European and North American cities [36]. Katoto *et al* [37] report that urban pollutant levels in SSA are often 10–20 times higher than WHO standards. Although far fewer studies have been conducted, noise pollution studies in South Africa [38] and Nigeria [39] show similar findings [40]. In Ethiopia, noise pollution from multiple sources in Wolaita Sodo city has also been documented to exceed WHO-recommended limits, with some measurements reaching double the tolerable decibel levels [41]. In Kigali, day and night-time noise levels often exceeded the Rwandan standard of 55 dBA for residential areas by over 60%, reaching more than ∼72% (day-time) and 80% (night-time) in some areas [42]. In Nyarugenge, the most urbanized district, noise levels exceeded day standards by as much as 75% [42].

We found that children attending school in Accra also have much higher exposure to PM_2.5_ at school than children in other major western cities such as New York and London. In New York City, PM_2.5_ levels ranged from 12 *µ*g m^−3^ in suburban schools to 23.1 *µ*g m^−3^ at schools in urban, secondary street settings [43]. A 2021 study found that the average PM_2.5_ concentration around schools in London was 12 *µ*g m^−3^ [44]. This further demonstrates potentially disproportionately higher exposures experienced by children in SSA cities compared to their counterparts in higher-income country cities, highlighting global environmental health inequities, though the evidence for comparisons is still limited.

Within-cities, evidence from around the world [45], and including from SSA cities, show that children from lower-resource communities may more often attend schools in areas with a higher density of pollution sources (e.g. proximity to major roads [46], high-density urban areas [47]), and thus resulting relatively higher levels of exposure [48, 49]. Our findings indicate that socioeconomic disparities in children’s exposure to air and environmental noise pollution are similarly present within Accra, where children living and learning in lower SES neighbourhoods experience higher levels of air and noise pollution, on average, than those in higher SES neighbourhoods, though variation within each group was still large. This pattern also persisted when comparing children attending public (free) schools compared with private (fee-paying) schools. The reasons underpinning these inequalities are varied, complex, and context dependent. Unlike the distinct zoning frameworks often seen in high-income countries, the urban exposome in SSA cities often features a highly localized, overlapping matrix of air pollution emission and acoustic sources that disproportionately burden marginalized communities [35]. Furthermore, traffic-related emissions (such as NO_2_ and BC) which have been found to be heavily concentrated around lower-SES areas in Accra, may be due to the city’s high reliance on aging, and poorly maintained commercial vehicle fleets and informal transit hubs (informal bus terminals or *tro tros*) that anchor poorer economic zones. Furthermore, household biomass burning is still common in Accra, particularly in lower income neighbourhoods [50]. Simultaneously, traffic congestion often coexists with community and neighbourhood noise sources, such as unregulated loudspeaker use for informal commercial activities [5], open-air religious activities [28], and street-side vendors, which are typically found in population dense, and lower SES areas of Accra, and other SSA cities [51].

Our data also showed an inverse relationship between neighbourhood SES and children’s exposure to nature-based sounds (animal and insect sounds) in the urbanised Accra core. Animals (e.g. birds) and insects are more prevalent in nature-based environments (i.e. spaces with trees, and other supporting habitats), which appear to be more common in higher SES areas of AMA, possibly due to larger residence compounds and private gardens. Furthermore, previous research in AMA found that average NDVI levels, a measure of the abundance of green vegetation, was 2-fold higher in areas in the highest SES quintiles compared with areas in the lowest [52]. Greenspace is also a natural noise attenuator with the ability to mask sounds. Notably, the soundscape literature suggests positive perceptions are associated with nature-based sounds, including perceived and actual restoration from stress and fatigue, inducing positive emotions [53], and restoring attentional capacity [54].

Beyond shaping differential exposures to air and noise pollution, socioeconomic inequalities may also influence the ability of households and schools to mitigate these exposures once present. In Accra, lower-income households may struggle to afford protective measures such as well-sealed building materials, double-paned windows, or indoor air purifiers [55], limiting their capacity to reduce indoor infiltration of outdoor pollutants and adapt the home to limit environmental risks [56]. The intersection of poverty and environmental exposure in Accra underscores the need for targeted mitigation efforts that prioritise vulnerable populations, particularly schoolchildren who spend a significant portion of their day, both at home and at school, in heavily polluted environments.

Another finding of our study concerns noise annoyance among children, a topic well-studied in adults in high-income countries but less so in children, particularly in LMICs. Research from high-income settings has consistently linked long-term environmental noise exposure, especially from transportation sources [57], to high annoyance (HA) in adults [58]. Our findings suggest that children in Accra show similar annoyance patterns in response to increasing noise levels, with potential implications for sleep [59], cognitive development [60], and academic performance [61]. The unadjusted exposure–response relationship between *L*_den_ levels at home and the percentage of highly annoyed (HA) children at home aligns broadly with international studies in adults: in our sample, ∼20% were HA at 60 dBA *L*_den_, rising to ∼35% at 65 *L*_den_ and ∼40% at 70+ *L*_den_. This mirrors global estimates by Fenech *et al* [62], which reported ∼15% HA at 60 dBA and 30%–50% between 70–80 dBA. However, like previous studies [63], Fenech *et al* [64], also highlight substantial regional variability, reinforcing the need for context-specific exposure–response functions. As part of the ASHES project, cognitive and academic performance metrics are being collected alongside noise exposure data, providing an opportunity to further examine these effects in future, providing more robust analyses of noise annoyance, controlling for confounders in the epidemiological models. Given the high levels of environmental noise found in Accra and the limited research on its long-term impacts in children, further study is both necessary and urgent.

*Strengths and limitations*

The ASHES study is among the first to simultaneously assess and characterize air and noise pollution in relation to socioeconomic inequalities among children in a major SSA city, providing a comprehensive view of multiple environmental stressors affecting child health at places where they spend the most time. We used data across multiple microenvironments to develop time-location weighted total exposure estimates, which improved accuracy and reduced exposure measurement error in future epidemiological studies. Further, in addition to the physical sound measurement, this study is among the first to examine the subjective experience/perception of noise among schoolchildren in SSA context, by looking at the unadjusted relationship between modelling environmental noise exposures and children’s reported annoyance to noise. Understanding this relationship in SSA, particularly amongst children, is important because noise annoyance is on the mechanistic pathway to a range of long-term chronic cardiometabolic [65], and mental health conditions [66]. With a sample of >900 schoolchildren from diverse socioeconomic backgrounds in a large metropolis, the findings are broadly applicable and generalizable within the context of children in Accra. Furthermore, the overarching ASHES study will leverage these geolinked air pollution and noise exposure estimates to conduct environmental epidemiological studies of child cardiovascular, sleep, and cognitive health in Accra in the future.

The ASHES study utilizes a total exposure metric that approximates children’s exposures by accounting for the average time they spend at two key locations (home and school). While the personal exposure metric represents an advancement in assessing children’s environmental exposures at this scale and in this context, it does not capture pollution levels in other locations where children may spend their time (e.g. in transit), nor does it reflect conditions inside their home or school buildings. Furthermore, the exploratory descriptive analysis of noise levels at home and children’s self-reported annoyance to noise was not adjusted for confounders. Thus, future research should employ more comprehensive statistical approaches for a more robust assessment of these exposure-response relationships. Future studies in Accra, but also more broadly across SSA cities, should also investigate greenspace exposures at home and school so that potential links between these and other exposures can be considered. Lastly, while the study offers valuable insights into environmental exposures in a rapidly urbanising SSA city, its findings may not be directly generalizable to rural settings or other SSA cities with different urban planning and pollution profiles.

## 5. Conclusion

This study provides critical new insights into the levels of air and environmental noise pollution and other urban sound metrics that children in a growing SSA city are exposed to at the places where they spend the most time. Our findings suggest that disparities in schoolchildren’s exposure to air and environmental noise pollution in Accra are not only shaped by school and home location but also by household and socioeconomic affluence, highlighting the intersection of environmental and socioeconomic factors in shaping exposure risks among urban children. Our findings have significant implications for environmental health policy, urban planning, and public health interventions in rapidly urbanizing SSA cities like Accra. By highlighting socioeconomic disparities in air and noise pollution exposure, the study underscores the urgent need for policies that prioritize environmental justice, to ensure that all children, regardless of SES, have access to healthier living and learning environments.

## Data Availability

The data cannot be made publicly available upon publication because they contain sensitive personal information. The data that support the findings of this study are available upon reasonable request from the authors. Supplementary Information 1 available at: https://doi.org/10.1088/1748-9326/ae8d40/data1.
